# Predicting Scenarios for Successful Autodissemination of Pyriproxyfen by Malaria Vectors from Their Resting Sites to Aquatic Habitats; Description and Simulation Analysis of a Field-Parameterizable Model

**DOI:** 10.1371/journal.pone.0131835

**Published:** 2015-07-17

**Authors:** Samson S. Kiware, George Corliss, Stephen Merrill, Dickson W. Lwetoijera, Gregor Devine, Silas Majambere, Gerry F. Killeen

**Affiliations:** 1 Environmental Health and Ecological Sciences Thematic Group, Ifakara |Health Institute, P.O. Box 53, Ifakara, Tanzania; 2 Department of Mathematics, Statistics and Computer Science, Marquette University, Milwaukee, WI, 53201–1881, United States of America; 3 QIMR Berghofer Medical Research Institute, Brisbane, Queensland, Australia; 4 Liverpool School of Tropical Medicine, Vector Biology Department, Pembroke Place, Liverpool, L3 5QA, United Kingdom; University of Crete, GREECE

## Abstract

**Background:**

Large-cage experiments indicate pyriproxifen (PPF) can be transferred from resting sites to aquatic habitats by *Anopheles arabiensis* - malaria vector mosquitoes to inhibit emergence of their own offspring. PPF coverage is amplified twice: (1) partial coverage of resting sites with PPF contamination results in far higher contamination coverage of adult mosquitoes because they are mobile and use numerous resting sites per gonotrophic cycle, and (2) even greater contamination coverage of aquatic habitats results from accumulation of PPF from multiple oviposition events.

**Methods and Findings:**

Deterministic mathematical models are described that use only field-measurable input parameters and capture the biological processes that mediate PPF autodissemination. Recent successes in large cages can be rationalized, and the plausibility of success under full field conditions can be evaluated *a priori*. The model also defines measurable properties of PPF delivery prototypes that may be optimized under controlled experimental conditions to maximize chances of success in full field trials. The most obvious flaw in this model is the endogenous relationship that inevitably occurs between the larval habitat coverage and the measured rate of oviposition into those habitats if the target mosquito species is used to mediate PPF transfer. However, this inconsistency also illustrates the potential advantages of using a different, non-target mosquito species for contamination at selected resting sites that shares the same aquatic habitats as the primary target. For autodissemination interventions to eliminate malaria transmission or vector populations during the dry season window of opportunity will require comprehensive contamination of the most challenging subset of aquatic habitats ${(C}_{l_{x}})$ that persist or retain PPF activity (*U*_*x*_) for only one week $(C_{l_{x}}\to 1$, where *U*_*x*_ = 7 days). To achieve >99% contamination coverage of these habitats will necessitate values for the product of the proportional coverage of the ovipositing mosquito population with PPF contamination (*C*_*M*_) by the ovitrap-detectable rates of oviposition by wild mosquitoes into this subset of habitats ${(m}_{l_{x,z,d}})$, divided by the titre of contaminated mosquitoes required to render them unproductive ${(T}_{l_{x,z,d}})$, that approximately approach unity $\left({C_{M}m}_{l_{x,z,d}}/T_{l_{x,z,d}}\to 1\right)$.

**Conclusions:**

The simple multiplicative relationship between *C*_*M*_ and $m_{l_{x,z,d}}/T_{l_{x,z,d}}$, and the simple exponential decay effect they have upon uncontaminated aquatic habitats, allows application of this model by theoreticians and field biologists alike.

## Introduction

The leading malaria vector control strategies (i.e., long-lasting insecticidal nets (LLINs) [1] and indoor residual spraying (IRS) [2]) can dramatically reduce transmission by mosquitoes that feed and/or rest indoors, but these tools alone are insufficient to eliminate it [3–6]. The success of LLINs and IRS rely on their ability to control mosquitoes that feed and/or rest indoors, so highly potent vectors like *Anopheles gambiae* and *An*. *funestus* that rely heavily upon human blood and houses for their survival can be effectively controlled or even eliminated by these approaches. However, the maximum impacts of LLINs and IRS are typically limited by the fact that many important primary vectors across the world [4–5, 7], and particularly *An*. *arabiensis* in the East African context [8–14], can evade exposure to them and survive by feeding upon animals or humans outdoors, or by avoiding contact with treated surfaces even when they do enter houses [15–17]. Hence, to achieve malaria elimination, strategies are needed that target mosquitoes when they feed on animals or humans outdoors, or while using one of the other biological and environmental resources they need, such as sugar, mating sites, resting sites, and oviposition sites [5, 18]. Some of the most promising strategies that might be used to complement LLINs and IRS by targeting adult mosquitoes outdoors include vapour-phase repellents [19], insecticide-treated clothing [20–21] insecticide-treated cattle [22], odor-baited traps [23–24] and toxic sugar baits [25]. Another, far older strategy, that has been used to suppress vector densities in a variety of contexts over the last century, is to prevent emergence of adults at source by applying insecticides to their aquatic larval habitats [26]. While this approach has achieved some striking successes against malaria vectors and transmission, even in Africa, its applicability and effectiveness may be limited by the substantial logistical challenges and associated costs of comprehensively and continuously identifying and treating relevant breeding habitats, especially in large rural areas with sparse human population [26–28].

However, it has proven possible to deliver larvicides by contaminating adult mosquitoes when they rest inside treated containers so that, when they subsequently oviposit, the insecticide is transferred to their aquatic larval habitats [29–31]. Such autodissemination of insecticide from resting sites to aquatic habitats via adult mosquitoes requires particularly potent larvicides, such as the pyriproxyfen (PPF) that interrupts normal development and metamorphosis of targeted mosquitoes [32–33]. PPF is a juvenile hormone analogue so only tiny amounts of active ingredient are required for it to be effective [34]. Success of this autodissemination strategy was first demonstrated for the ideally-suited dengue vector, *Aedes aegypti*, which breeds in sealed containers that retain PPF and protect it against extremes of temperature and solar radiation [29–31].

More recently, autodissemination of PPF by the malaria vector *Anopheles arabiensis* has been demonstrated in large-cage SFS experiments [35], but it remains to be seen whether similar levels of success can be achieved with field populations under natural conditions. It is essential to understand quantitatively the distinct processes that drive the autodissemination phenomenon, so that prototypes for implementing this strategy can be designed, optimized, and evaluated. Unlike the container-breeding *Aedes* species that PPF autodissemination has been so effective against under full field conditions [29–31], most aquatic habitats of *An*. *arabiensis* and other members of the *An*. *gambiae* complex are extremely dynamic in hydrological terms [36–39]. This approach is therefore least likely to be effective in the wet season when most malaria transmission occurs, but most likely to be effective in the dry season when malaria parasites and vectors go through their annual seasonal population bottleneck [6, 40–41]. The most obvious challenge, which autodissemination shares with conventional manual application of larvicides, is that most of the many habitats available during the rains are far more ephemeral and have higher rates of hydrological turnover than the far smaller subset that persist throughout the dry season, because they are supported by dynamic rainfall and runoff inputs, rather than only stable groundwater reservoirs [36–39]. However, unlike conventional manual larvicide application, autodissemination relies upon adult mosquitoes to deliver the active ingredient, so effectiveness is expected to vary with the ratio of adult mosquito density to available aquatic habitat. These target vector species proliferate opportunistically in a wide diversity of ephemeral habitats, the available quantity of which fluctuates wildly over the course of the wet season in response to sporadic dry and wet periods [36, 39]. In the early stages of seasonal population surges in response to rainfall, the amount of habitat often increases even faster than the spectacular maximum population growth rates of these species [42–44]. The size of adult vector populations, relative to the carrying capacity of available aquatic habitat, therefore reach seasonal minima during and immediately after periods of rainfall, with observable maternal effects upon body size of subsequent generations that benefit from lack of competition for aquatic habitats [42–44]. Therefore, the periods of maximal vector population growth that are most important to year-round control of malaria transmission are the least suited to attack with PPF autodissemination and may well be intractable. By contrast, after the rains have ended and adult populations slowly contract to the minimum size that remaining dry season habitats can sustain, larval competition and, presumably, oviposition input per unit of habitat are maximal and may exceed carrying capacity [42–44], thus creating optimal conditions for success of PPF autodissemination. Such a dry season optimum may seem discouraging because most annual vector proliferation and malaria transmission obviously arises during the wet season. However, it does create an opportunity to implement a seasonally-targeted supplementary intervention, to eliminate local malaria parasite and vector populations when they are smallest and most vulnerable [6, 40–41], and the hydrological systems supporting both the aquatic habitats and the vector populations approach steady-state conditions [38].

Autodissemination of PPF has been previously described using a steady-state mathematical model [45] that crudely describes the relationship between the effective coverage of adult resting sites (*C*_*r*_) and larval habitats (*C*_*h*_) with PPF contamination, using a simple exponential model of PPF accumulation and decay based on the time (in days or nights) over which contaminated habitats persist but remain unproductive (*U*), the total number of ovipositions (*O*) by the entire adult population per night, the number of larval habitats (*H*), and the number of contaminating events needed to make a single habitat unproductive (Ω):

$$C_{h}=1-e^{\frac{-C_{r}UO}{ΩH}}.$$ (1)

While the mean time period over which contaminated aquatic larval habitats persist but remain unproductive (*U*) may, in principle, be measured by direct observation of habitat persistence and sampling of pupae or emerging adults, it is difficult to define and impossible to measure absolute values for the input parameters *C*_*r*_, *H*, *O*, and Ω as originally defined [45] for several reasons: (1) The absolute proportion of all resting sites that have been contaminated with PPF (*C*_*r*_) is inestimable because it is not feasible to define measurable units for all forms of resting sites, much less survey them [46–47]; (2) The number of hydrologically independent habitats (*H*) cannot be quantified simply because it is not practically possible to define clearly, much less measure, what constitutes genuine larval habitat, as opposed to the water bodies with which they are associated [36, 38–39, 47]. Absolute quantities of habitat, expressed in terms of volume or area are fundamentally un-measurable for a species like *An*. *arabiensis* because they are poorly defined and dynamic, often consisting of tiny depressions at the edges of water bodies or vegetation-sheltered peripheral portions within them [36, 38–39]. For example, the absolute sizes of discrete habitats, like puddles, footprints and stands of emergent vegetation, around the fringe of a large lake may be comparable to those around a small pond, but many of these are essentially impossible to identify and delineate unambiguously; (3) the number of ovipositions carried out each night by the entire population across all habitats (*O*) or even per habitat in a sample of habitats (*O*/*H*) cannot be quantified because the only existing trap for capturing free-flying, gravid, wild *Anopheles*, when they oviposit in natural aquatic habitats, only samples unknown fractions of the total number of ovipositing females visiting those habitats [48], which are themselves impossible to distinguish and quantify [36, 38–39, 47]. While other prototype oviposition traps for *Anopheles* do exist, they may be applied only to artificial or modified sentinel habitats [49–50]; (4) The number of contaminating events needed to make even a single, selected habitat unproductive Ω cannot be estimated because, as described for (*H*), naturally-occurring habitats are extremely difficult to define, distinguish and quantify unambiguously [36, 38–39, 47]. Also, performing titration by introducing varying numbers of contaminated mosquitoes into such habitats within cages placed over them do not necessarily correspond to equivalents number of contamination events because a single mosquito may oviposit more than once [51]. In addition, those contamination events are themselves impossible to quantify with existing oviposition traps for the same reasons as (*O*/*H*).

**Fig 1:**
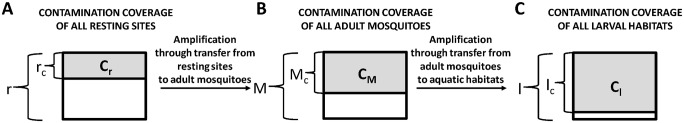
A schematic illustration of how partial coverage all resting sites is amplified in two steps as PPF contamination is transferred to the adult mosquito population and then onwards to the larval habitats. The proportional coverage of the resting sites (*C*_*r*_), ovipositing adult mosquito population (*C*_*M*_) and larval habitats (*C*_*l*_) is depicted as a proportion of all resting sites (*r*), adult mosquitoes (*M*) and larval habitats (*l*) covered with PPF contamination (*c*).

Furthermore, recent large-cage SFS experiments with *An*. *arabiensis* [35] clearly demonstrate that autodissemination via this vector species, which is behaviorally resilient to control with LLINs or IRS [8–17], actually involved two coverage amplification steps (Fig 1), rather than merely one as previously assumed [45]. The previous formulation [45] assumed that the proportional coverage of resting sites (*C*_*r*_) with PPF contamination and of the ovipositing adult mosquito population (*C*_*M*_) are equivalent, and that coverage amplification occurs through a single process as PPF is accumulated in larval habitats through repeated transfer from contaminated adults. However, these large-cage experiments [35] demonstrate how coverage amplification also occurs as PPF is transferred from the resting sites to the mosquito population (Fig 1): Taking the proportion of all sampled mosquitoes that were recovered from clay pots as a crude indicator of resting site coverage (*C*_*r*_ = 0.17) and contrasting this with the high proportion of mosquitoes caught outside of the pots that were contaminated (*C*_*M*_ = 0.72), illustrates how an approximately four-fold amplification (*C*_*M*_/*C*_*r*_ = 0.72/0.17 = 4.2) apparently occurred. This additional amplification step presumably occurs because mosquitoes move around through several resting sites over the course of a night, as demonstrated by the direct observation of such high proportions of contaminated mosquitoes outside of the treated pots.

Here, we revise and reformulate the previously published model [45] and adapt some formulations from another more recent model that allows multiple resource utilization events per gonotrophic cycle for resting sites or other resources to be measured and accounted for [46], so that a range of alternative approaches to implementing such a double-amplification autodissemination strategy can be parsimoniously modelled using only input parameters that are field-measurable. Simulation analysis explores conditions under which an autodissemination of insecticide strategy might be successful in the field.

## Methods

Since the publication of the model described in Eq 1 [45], we have developed a broader set of generalizable models for capturing the effects of a wide variety of intervention strategies that target diverse resources which mosquitoes utilize, such as sugar, mating sites, resting sites, and oviposition sites, as well as subsets of those resources [3, 16, 23, 46, 52–54]. Here we adapt the notations and definitions of the previous autodissemination model [45] to harmonize them with these broadly applicable frameworks and to enable development into a far more explicit, practically applicable, and field-parameterizable form. In particular, the notations and definitions are revised to enable the modelling of a range of alternative approaches to the autodissemination strategy in a way that explicitly captures the changing levels of coverage achieved as PPF is transferred, first from one of several possible resource subsets that could act as targets for initial delivery to the adult mosquito population via contact contamination, and then from those adult mosquitoes to the ultimate aquatic habitat targets when they make contact by oviposition (Fig 1). First of all, the notation is adjusted (Table 1) by (1) substituting *C*_*M*_ for *C*_*r*_ to reflect direct dependence of the proportional coverage of all aquatic larval habitat (*l*) with PPF contamination (*C*_*l*_) upon that of the ovipositing adult mosquito population (*C*_*M*_) and only indirectly upon that of the resting sites (*C*_*r*_), (2) substituting *T*_*l*_ for Ω to reflect potential for measurement by mosquito exposure titration experiments and to prevent overlap in meaning with previous uses of the symbol Ω to reflect distinct vector control scenarios [3, 16, 23, 52–54], and (3) substituting *l* for *H* to enable consistent use, not only as a subscript to specify the proportional coverage of all aquatic larval habitat (*l*) with PPF contamination (*C*_*l*_), but also as all forms of that entire specific resource (*R* = *l*) [46] as illustrated in Fig 1. Eq 1 is therefore reformulated as

$$C_{l}=1-e^{-\;\frac{C_{M}\;U\;O}{\;T_{l}\;l}\;}.$$ (2)

**Table 1:** Parameter symbols and definitions.

Symbol	Definition
*α*_*r*_	Utilization rate for an entire given resting site defined as the rate at which individual mosquitoes attempt to utilize all forms of that resting site per gonotrophic cycle
${\alpha_{r}}_{x}$	Utilization rate for a defined subset of a given resting site, defined as the rate at which individual mosquitoes attempt to utilize the subset (*x*) per gonotrophic cycle
*α*_*l*_	Mean utilization rate of all aquatic larval habitats (*l*) by individual mosquitoes defined as the rate at which individual mosquitoes attempt to utilize any aquatic larval habitat per gonotrophic cycle
*C*_*r*_	Proportional coverage of all resting sites (*r*) with PPF contamination
$C_{r_{x}}$	Proportional coverage of all available forms of an identifiable, targetable subset (*x*) of a resting site (*r*) with PPF contamination
*C*_*l*_	Proportional coverage of all aquatic larval habitats (*l*) with PPF contamination
$C_{l_{x}}$	Proportional coverage of an identifiable, targetable subset (*x*) of of all available forms of aquatic larval habitat (*l*) with PPF contamination
*M*	Absolute size of the mosquito population in a given setting, defined in terms of the number of individuals present
$m_{r_{x,z}}$	Rate at which the mosquito population utilizes a surveyed sample subset (*z*) of any identifiable and targetable subset (*x*) of resting sites (*r*_*x*,*z*_), expressed as the number of utilization attempt events per night
$m_{v_{z}}$	Rate at which the mosquito population utilizes a defined, entomologically surveyed sample (*z*) of blood resources (*v*_*z*_), expressed as the number of utilization attempt events per night
*T*_*l*_	The minimum rate at which contaminated ovipositing females oviposit into all aquatic larval habitats in an ecosystem (*l*) that is required to render those habitats unproductive within one night
$T_{l_{x,z,d}}$	The minimum rate at which contaminated ovipositing females are captured by sticky traps placed at a defined density per unit of habitat perimeter length at a given sample (*z*) of a given subset (*x*) of all available forms of aquatic larval habitat (*l*_*x*,*z*_), that is required to render those habitats unproductive within one night
$m_{l_{x,z,d}}$	Rate at which the mosquito population utilizes a defined, entomologically surveyed sample (*z*) of subset (*x*) of aquatic larval habitat (*l*) as detected (*d*) by sticky traps placed at a defined density per unit of habitat perimeter length at, expressed as the number of utilization attempt events per night.
*U*	Mean time period that all aquatic larval habitats, or subset (x) of those habitsts (*U*_*x*_), persist but remain unproductive following contamination with PPF.
*ε*_*d*_	Detection efficiency of a given capture method (in this case, sticky traps for capturing mosquitoes when they oviposit) or observational method defined as the proportion of events occurring within the surveyed subset of habitats over the surveyed period that are detected
*C*_*M*_	Proportional coverage of the ovipositing adult mosquito population (*M*) with PPF contamination
$\mu_{R_{c}}$	Mortality probability associated with exposure to an intervention-covered (*c*) form of a given resource (*R*) through a single utilization attempt event
$P_{\alpha_{R_{c}}}$	Probability of a mosquito surviving all attempts to utilize intervention-covered (*c*) forms of the targeted resource (*R*) per gonotrophic cycle
*r*	The total availability of all forms of resting sites, defined as the rate at which individual mosquitoes encounter and attempt to utilize resting sites per night
*l*	The total availability of all forms of aquatic larval habitats, defined as the rate at which individual mosquitoes encounter and attempt to utilize aquatic habitat per night
*v*	The total availability of all forms of blood, defined as the rate at which individual mosquitoes encounter and attempt to utilize blood per night
*X*	A subset of a given resource that may be identified and targeted with a vector control intervention
*z*	A sample of a given resource that has been surveyed entomologically
*O*	The ecosystem-wide total nightly oviposition rate by the entire adult mosquito population into all aquatic larval habitats or into a subset (*x*) of those habitats (*O*_*x*_).

In the following sections, we describe how each of the parameters in Eq 2 have been conceptually redefined and may be estimated in the field.

### 2.1 Amplification of contamination coverage through transfer of pypriproxifen from treated resting sites to adult mosquitoes

We have revised the definition of the coverage term on the right hand side of Eq 1 to represent more accurately its original conceptual basis. This coverage term was originally and mistakenly described as the proportional coverage of all resting sites with PPF contamination (*C*_*r*_) [45], but the conceptual basis of the equation is that it describes the proportional coverage of the ovipositing adult mosquito population (*M*) with PPF contamination (*C*_*M*_). Therefore, the original formulation implicitly assumed that *C*_*r*_ are *C*_*M*_ are equivalent (*C*_*M*_ ≈ *C*_*r*_) because each mosquito rested in only one location per gonotrophic cycle. The assumption that a mosquito visits only a single resting site in a typical full gonotrophic cycle is obviously questionable for many mosquito species [55]. Furthermore, recent observations during experiments to demonstrate autodissemination of PPF by *An*. *arabiensis* specifically, indicate that a high proportion of mosquitoes caught outside the treated pots appeared to be contaminated with this larvicide [35] clearly demonstrate just how inaccurate this assumption is in relation to this specific mosquito species. Thus, we introduce an additional, intermediate parameter which describes coverage of the entire adult mosquito population (*M*) that mediates autodissemination of PPF. It is important that an approach used to measure the proportional coverage of adult mosquito population contaminated with PPF can account for all contaminating resting sites visited by a mosquito per gonotrophic cycle. Fortunately, several techniques such as more sensitive chemical, biochemical, genetic and biological markers [56] may be used to label mosquitoes directly when they are resting. Therefore, proportional coverage of the ovipositing adult mosquito population (*M*) with PPF contamination (*C*_*M*_) may then be measured directly by testing samples of individual mosquitoes for PPF contamination or its biological activity [35], or alternatively with a variety of markers [56] that can be used as more convenient, readily-detected surrogates for PPF contamination.

Nevertheless, while measurements of *C*_*M*_ using such labels are essential and more direct predictors of autodissemination success, it may also be estimated indirectly from entomological surveys of the resting site utilization processes that directly mediate it. Furthermore, to fully understand how two-stage autodissemination strategies function by measuring the rate at which PPF-targeted subsets (*x*) of resting sites (*r*) are utilized $\left(\alpha_{r_{x}}\right)$, and to cross-validate these measures by comparison with each other, it is important to also model the first coverage amplification process as PPF is transferred from those resting sites to the adult mosquito population (Fig 1).

In this revised formulation, proportional coverage of the ovipositing adult mosquito population (*M*) with PPF contamination (*C*_*M*_) that mediates transfer from the treated resource to the aquatic larval habitat resource (*l*) is assumed to be a function of the rate at which all resting sites that are covered with PPF contamination (*r*_*c*_) are visited by mosquitoes ${(\alpha }_{r_{c}})$, which is in turn the product of the coverage of all available contaminated and uncontaminated resting sites [46] (*C*_*r*_) and the rates at which individual mosquitoes utilize all available resting site surfaces *α*_*r*_:

$$C_{M}=f(\alpha_{r_{c}})=f(\;\alpha_{r},C_{r})\;.$$ (3)

The latter utilization rate term is defined as the mean number of times an individual mosquito makes physical contact with any contaminated or uncontaminated resting site surface during a typical gonotrophic cycle. Hence, instead of assuming the proportion of all contaminated adult mosquitoes is approximately equivalent to the proportion of all available contaminated resting sites (*C*_*M*_ ≈ *C*_*r*_), we present an exponential relationship relating proportional coverage of the ovipositing adult mosquito population (*M*) with PPF contamination (*C*_*M*_) to coverage (*C*_*r*_) and utilization rate (*α*_*r*_) of all available resting sites (*r*), rather than just those that have been covered with PPF contamination (*r*_*c*_). Also, the new terms for the per gonotrophic cycle utilization rate of a resource (*α*_*R*_) or resource subset ${(\alpha }_{R_{x}})$ also is previously introduced [54], so that the effects of covering resources that may be utilized more than once per gonotrophic cycle can be modelled [46].

Even assuming that the proportional coverage of the ovipositing adult mosquito population (*M*) with PPF contamination (*C*_*M*_) is a function of proportional coverage of all resting sites (*r*) with PPF contamination (*C*_*r*_) and the utilization rate for all resting sites (*α*_*r*_), the problem remains that neither can be reliably measured, even within the confines of our SFS because it is impossible to quantify or survey all the possible surfaces mosquitoes may choose to rest upon.

Fortunately, in cases where the total amount of a given mosquito resource (*R*) (such as blood (*v*), resting sites (*r*), or aquatic habitat (*l*)) cannot be quantified, it is possible to predict the impact of conventional insecticides that directly kill adults based on the measurements of coverage $\left(C_{R_{x}}\right)$ and utilization rates $\left(\alpha_{R_{x}}\right)$ for any definable, targetable subset (*R*_*x*_) of that overall resource [46]. The advantage of using $\alpha_{R_{x}}$ and $C_{R_{x}}$ is that both are directly measurable. It is no longer necessary to know the proportion of the total resource which the covered subset represents (*C*_*R*_) or the utilization rate for all available forms of that resource (*α*_*R*_). Specifically, the product of the proportional coverage of all resting sites (*r*) with PPF contamination (*C*_*r*_) and utilization rate (*α*_*r*_) of all resting site is equivalent to the product of the corresponding terms for the insecticide-targeted subset ($C_{r_{x}}$ and $\alpha_{r_{x}}$, respectively), which are both field-measurable parameters for such quantifiable, surveyable, subsets of resting sites [46],

$$\alpha_{r_{c}}=C_{r}\alpha_{r}=\alpha_{r_{x}}C_{r_{x}}\;.$$ (4)

A previous formulation in [46] designed to predict mosquito mortality resulting from resting surfaces treated with insecticide that kill them on contact was adapted to predict proportional coverage of the ovipositing adult mosquito population (*M*) with PPF contamination (*C*_*M*_) by substituting the term contamination for mortality. In this preceding formulation [46], the probability of surviving all attempts to use intervention-covered forms of the targeted resource, in this case specified as resting sites (*R* = *r*) per gonotrophic cycle ($P_{\alpha_{r_{c}}}$), is calculated as a simple exponential decay function of the product of the mortality probability associated with exposure to a covered form of the resting site through a single utilization event ($\mu_{r_{c}})$ and the mean utilization rate for all covered forms of that resting site $\left(\alpha_{r_{c}}\right)$ [46] which may be substituted with the product of the proportional coverage of all available forms of an identifiable, targetable subset (*x*) of a resting site (*r*) with PPF contamination ${(C}_{r_{x}})$ and utilization rate for a defined subset of resting sites $(\alpha_{r_{x}}$) from Eq 4,

$$P_{\alpha_{r_{c}}}=e^{-\mu_{r_{c}}\alpha_{r_{c}}}=e^{-\mu_{r_{c}}\alpha_{r_{x}}C_{r_{x}}}.$$ (5)

By definition, $P_{\alpha_{r_{c}}}$ also may be understood as the probability per gonotrophic cycle of an individual mosquito of not being killed through contact with insecticide-covered forms of a targeted resource. This complementary definition can be adapted readily to calculate the probability of adult mosquitoes not being contaminated with PPF. Hence, replacing the mortality term with the probability of mosquito contamination resulting from a single exposure to a PPF-contaminated resting site through a single utilization event $\left(\zeta_{r_{c}}\right)$, and then replacing the survival probability term $\left(\rho_{\alpha_{r_{c}}}\right)$ with the probability per gonotrophic cycle of not being contaminated with PPF through contact with any of the covered resting sites, we get the equivalent formulation

$$\rho_{\alpha_{r_{c}}}=e^{-\zeta_{r_{c}}\alpha_{r_{x}}C_{r_{x}}}.$$ (6)

Therefore, the proportional coverage of the ovipositing adult mosquito population (*M*) with PPF contamination is the complement of the probability of not being contaminated with PPF,

$$C_{M}=1-\rho_{\alpha_{r_{c}}}=1-e^{-\zeta_{r_{c}}\alpha_{r_{x}}C_{r_{x}}}.$$ (7)

In addition, the contaminating probability associated with exposure to a PPF-contaminated form of the resting site through a single utilization event may be reasonably assumed to approach unity $\left(\zeta_{r_{c}}\to 1\right)$ based on experimental data indicating that 100% of all mosquitoes caught resting within a clay pot treated with PPF are contaminated [35], so

$$C_{M}\approx 1-e^{-\alpha_{r_{x}}C_{r_{x}}}.$$ (8)

Hence, the proportional coverage of the ovipositing adult mosquito population (*M*) with PPF contamination can be calculated directly using only two field-measurable parameters for the targetable, quantifiable, surveyable subset, specifically the proportional coverage of all available forms of that subset (*x*) resting sites (*r*) with PPF contamination ${(C}_{r_{x}})$ and the population mean utilization rate for that resting site subset by individual mosquitoes $\left(\alpha_{r_{x}}\right)$.

While $C_{r_{x}}$ may be readily and rapidly surveyed by direct inspection, $\alpha_{r_{x}}$ may be estimated indirectly by comparison with the rate at which the mosquito blood feeding events occur at the population level [46]. Otherwise, it is impossible to quantify directly the rates at which mosquitoes make contact with a subset of resting sites which is definable and measurable in itself but constitutes an unknown fraction of an indefinable, un-measurable total quantity of resting sites [46]. By comparison, numbers of blood hosts of particular species can be readily quantified, as can the rates at which mosquitoes blood feed upon them and the proportion of all blood meals that each host species represents, so it is possible to estimate the rate at which blood meals are taken or gonotrophic cycles are completed by a mosquito population (*m*_*v*_) or population sample ${(m}_{v_{z}})$ [46]. Thus, if the rate at which a defined, targetable subset of resting site resources (*r*_*x*_) are visited by the same mosquito population $\left(m_{r_{x}}\right)$ or population subsample $\left(m_{r_{x,z}}\right)$ also can be estimated, the mean rate at which individual mosquitoes visit that resting site subset per gonotrophic cycle may be calculated as the quotient of these two quantities [46]:

$$\alpha_{r_{x}}=\frac{m_{r_{x}}}{m_{v}}=\frac{m_{r_{x,z}}}{m_{v_{z}}}\;.$$ (9)

In practice, $m_{r_{x,z}}$ may be measured by either capturing or observing mosquitoes when they rest at samples (*z*) of the targeted resting site subset (*x*) [46]. Also, $m_{v_{z}}$ may be measured by capturing mosquitoes when they attack samples of humans or livestock and dividing by the product of the proportion of all hosts of that species that sample accounts for and the proportion of all bloodmeals that species accounts for, as described in detail elsewhere [46]. Note, however, that estimating $\alpha_{r_{x}}$ in this indirect manner requires local measurement of several entomological input parameters that are laborious and imprecise so this approach will most probably be far less direct, precise and accurate than direct labeling of mosquito populations to measure *C*_*M*_ [46].

In fact, even if it is useful to measure the target resting site subset utilization rate ($\alpha_{r_{x}}$) in addition to mosquito population coverage, it is probably far easier and more accurate to estimate the former as a simple function of measured values for the latter. Eq 8 may be rearranged so that *α*_*r*,*x*_ can be calculated either directly from single measurements of *C*_*M*_ or estimated by fitting the following equation to measures of *C*_*M*_ at varying levels of coverage of the targeted resting site subset ($C_{r_{x}}$):

$$\alpha_{r_{x}}=-\frac{\text{ln}(1-C_{M})}{C_{r_{x}}}.$$ (10)

### 2.2 Amplification of contamination coverage through transfer of PPF from gravid adult mosquitoes to aquatic habitats

The four other parameters in Eq 2, namely the ecosystem-wide total nightly oviposition rate by the entire adult population (*O*), the mean time period that aquatic larval habitats persist but remain unproductive following contamination with PPF (*U*), the total availability of all forms of aquatic larval habitats (*l*), and the mean number of contaminating oviposition events needed to make a single habitat unproductive (*T*_*l*_), all relate to transfer and accumulation of PPF in aquatic habitats. However, as originally defined, these are not practically measurable in the field, so these components also are revised at a fundamental conceptual level.

#### 2.2.1 Calculating the minimum number of ovipositions by contaminated mosquitoes required to render habitats unproductive

The minimum rate at which contaminated ovipositing females oviposit into all aquatic larval habitats in an ecosystem (*l*) that is required to render those habitats unproductive within one night (*T*_*l*_) can be measured in a large-cage SFS with one or more artificial habitats by simple titration. In principle, such a titration measurement may be accomplished by measuring the impact of PPF delivered by varying numbers of released, contaminated mosquitoes. The term Ω in the original model [45] is therefore replaced by (*T*_*l*_) to reflect that titration measurement opportunity and avoid conflicting with previous models using the former symbol to denote vector control scenario [23, 52–53]. The mean titre of all habitats (*T*_*l*_) is defined as the minimum rate at which contaminated females oviposit into them per night that is required to reach a targeted percentage (usually ≥ 95%, but ≥ 99% is more appropriate for such a strategy intended to eliminate rather than merely control vector populations [6] and the malaria transmission they mediate [40]) of emergence inhibition of adult mosquitoes from contaminated aquatic larval habitats. Mathematically, *T*_*l*_ may be calculated as the product of the mean utilization rate of all aquatic larval habitat(s) by individual mosquitoes (*α*_*l*_) and the minimum rate at which contaminated females must oviposit into them to render those habitats unproductive within one night $\left(m_{l}^{min}\right)$, divided by the total quantity of those aquatic larval habitats (*l*),

$$T_{l}=\alpha_{l}\frac{m_{l}^{min}}{l}.$$ (11)

However, the overall total oviposition event titre for all aquatic larval habitats present in any natural ecosystem (*T*_*l*_) is impossible to measure in practice. Furthermore, titre estimates for artificially constructed habitats are of dubious relevance to natural habitats, which are far more diverse, dynamic, and variable in qualitative and quantitative terms [36, 39]. In principle, titration experiments could be conducted in natural habitats in the field by temporarily placing large cages over them and releasing varying numbers of contaminated, insectary-reared gravid females. However, the obstacle that remains to predicting impact of an autodissemination strategy is estimating the natural rates of exposure of these habitats to ovipositing females in the absence of any way to measure the total number of ovipositing mosquitoes visiting them or the total quantity of habitat surveyed.

Fortunately, a recently developed method [48] for surveying oviposition contacts of mosquitoes with either artificial or natural aquatic larval habitats, by trapping them on glue-covered plastic sheets, now allows an index of oviposition input to be recorded. This method probably exhibits incomplete efficiency of oviposition contact detection (*ε*_*d*_) through physical capture (*ε*_*d*_<1), but the number of oviposition events that can be observed with this sticky trap method in a given habitat sample (*z*) for a given titration experiment can be assumed to be proportional to total oviposition contacts if that efficiency level is consistent for each habitat type (*x*) category, such as puddles, river fringes, or springs and the sticky traps are placed at a fixed density per unit of habitat perimeter:

$$T_{l_{z,d}}=\varepsilon_{d}T_{l_{z}},$$ (12A)

and

$$T_{l_{x,z,d}}=\varepsilon_{d}T_{l_{x,z}}.$$ (12B)

Failures of the trap to capture mosquitoes that make contact with it may lead to incomplete trapping of all mosquitoes visiting a habitat. More crucially, however, each trap only surveys a sample of the perimeter of any water body where most larval habitat occurs. This is an advantage because it presents a valuable opportunity to field-parameterize these models. While it is not possible to estimate which fraction of all larval habitat (*l*) or subset thereof (*l*_*x*_) that any given set of sticky oviposition traps (*l*_*z*_ or *l*_*x*,*z*_ respectively), represent, it can be assumed to vary in proportion to the rate of oviposition input per *unmeasurable but constant* unit of quantity of habitat such traps are considered to sample (*l*_*z*_ or *l*_*x*,*z*_ respectively), regardless of how much unknown, un-measurable total habitat (*l*) or habitat subset (*l*_*x*_) is present in the ecosystem. As described in detail below, the absolute titre estimates for samples of natural habitats can be replaced by titres of detected (*d*) oviposition events at samples (*z*) per unit of perimeter length at habitats or subsets (*x*) of habitats, measured with oviposition sticky traps [48]. This new term for the detectable oviposition contact titre $(T_{l_{z,d}}\;or\;T_{l_{x,z,d}}\;$ respectively), is expressed as the minimum rate at which contaminated ovipositing females are captured by sticky traps placed at a defined density per unit of habitat perimeter length at a sample of aquatic habitat that is required to render those habitats unproductive within one night.

Consider an SFS or full field experiment undertaken to measure the minimum number of ovipositing mosquitoes utilizing the habitat(s) that are required to render it unproductive ${(m}_{l}^{min})$ and the total quantity of habitat (*l*) where sticky traps [48] are used to measure an index of the number of oviposition events by mosquitoes for a sample (*z*) of a categorical subset (*x*) of aquatic habitats (*l*_*x*,*z*_). It is assumed that the numbers of mosquitoes caught by a single sticky trap represent a detectable fraction (*ε*_*d*_) of all utilization events occurring at the unmeasurable but constant unit of habitat each one can cover by placing them at a defined density per unit of perimeter length ${(m}_{l_{x,z}})$, which is typically distributed along the perimeter of water bodies rather than in them, because 1) they are applied at a constant density per unit of perimeter in existing protocols, and 2) each sticky trap has a fixed area and dimensions [48]. If $m_{l_{x,z,d}}^{min}$ represents the mean minimum catch per night per sticky trap that results in lack of productivity following controlled exposure to contaminated mosquitoes, then the detectable oviposition titre of detected oviposition events per night per sticky trap ($T_{l_{z,d}}$ or $T_{l_{x,z,d}}$) may be computed as

$$T_{l_{z,d}}=\alpha_{l}\frac{m_{l,z,d}^{min}}{l_{z}}=\alpha_{l}\varepsilon_{d}\frac{m_{l,z}^{min}}{l_{z}},$$ (13A)

or

$$T_{l_{x,z,d}}=\alpha_{l}\frac{m_{l_{x,z,d}}^{min}}{l_{x,z}}=\alpha_{l}\varepsilon_{d}\frac{m_{l_{x,z}}^{min}}{l_{x,z}}\;,$$ (13B)

respectively, where $m_{l_{z}}$ of $m_{l_{x,z}}$ is the number of oviposition events occuring in the aquatic habitats or habitat subset that was surveyed with the sticky traps, and *ε*_*d*_ is the detection sensitivity of those events by the sticky trap.

Assuming that the sample of all aquatic larval habitats (*l*_*z*_) or a categorical subset thereof (*l*_*x*,*z*_) is representative, it also may be assumed that the mean catch per night per sticky trap for a sample of that habitat (*l*_*z*_) or habitat subset (*l*_*x*,*z*_) is also representative of the mean catch per night per sticky trap for the entire set (*l*) or subset (*l*_*x*_) of habitats, and therefore, proportional to the fraction of all aquatic habitats that surveyed samples represent:

$$\frac{m_{l_{z}}^{min}}{m_{l}^{min}}=\frac{l_{z}}{l},$$ (14A)

and

$$\frac{m_{l_{x,z}}^{min}}{m_{l_{x}}^{min}}=\frac{l_{x,z}}{l_{x}}.$$ (14B)

Re-arranging Eq 13 yields

$$\alpha_{l}\frac{m_{l_{z}}^{min}}{l_{z}}=\frac{T_{l_{z,d}}}{\varepsilon_{d}},$$ (15A)

and

$$\alpha_{l}\frac{m_{l_{x,z}}^{min}}{l_{x,z}}=\frac{T_{l_{x,z,d}}}{\varepsilon_{d}}.$$ (15B)

Re-arranging Eq 14 yields

$$\frac{m_{l}^{min}}{l}=\frac{m_{l_{z}}^{min}}{l_{z}},$$ (16A)

and

$$\frac{m_{l,x}^{min}}{l_{x}}=\frac{m_{l_{z,x}}^{min}}{l_{x,z}}.$$ (16B)

Substituting Eq 16 and then Eq 15 into Eq 11 yields

$$T_{l}=\alpha_{l}\frac{m_{l}^{min}}{l}=\alpha_{l}\frac{m_{l_{z}}^{min}}{l_{z}}=\frac{T_{l_{z,d}}}{\varepsilon_{d}}.$$ (17A)

and

$$T_{l,x}=\alpha_{l}\frac{m_{l_{x}}^{min}}{l_{x}}=\alpha_{l}\frac{m_{l_{x,z}}^{min}}{l_{x,z}}=\frac{T_{l_{x,z,d}}}{\varepsilon_{d}}.$$ (17B)

Hence, even without knowing total availability of all forms of aquatic larval habitats (*l* or *l*_*x*_) or the utilization rate of oviposition sites by individual mosquitoes per gonotrophic cycle $\left(\alpha_{l}\;or\;\alpha_{l_{x}}\right)$, in principle, the absolute titre of all habitats may be calculated by dividing the known detectable titre of the sampled habitats $\left(T_{l_{z,d}}\;or\;T_{l_{x,z,d}}\right)$ by the detection efficiency of the sticky trap (*ε*_*d*_). It is not obvious how the detection efficiency of the sticky trap could be measured, except perhaps by direct observation [57–58]. However, as explained below, it is not essential to know the absolute oviposition input titre so long as the titration experiments use the same imperfect sampling tool as surveys of oviposition exposure of the same natural habitats to wild mosquito populations. Mathematically, this allows a fully measurable solution to Eq 2 because, as described in the next sub-section, the unmeasurable detection sensitivity term (*ε*_*d*_) also appears in the otherwise fully measurable solution to the quotient *O*/*l*, or an equivalent term in a model for a defined subset of habitats *O*_*x*_/*l*_*x*_.

#### 2.2.2 Calculating the ratio between the numbers of ovipositions by adult mosquitoes and aquatic habitats

The remaining terms to be addressed include only the ecosystem-wide total nightly oviposition rate by the entire adult population (*O*), and the quantity of aquatic larval habitats available for them to oviposit into (*l*), which constitute a quotient (*O*/*l*) in Eq 2.

Unfortunately, the mean number of oviposition events each gravid mosquito executes per gonotrophic cycle remains unknown but clearly greater than unity for African *Anopheles* studied thus far (*α*_*l*_>1) [51]. It is therefore not possible to measure this utilization rate for oviposition sites directly, or to reliably infer the population-level total rate at which these events occur in an entire ecosystem, even if the total rate at which mosquitoes become gravid and begin ovipositing (*m*_*l*_) could be inferred from estimates of gonotrophic cycle completion based on surveys of blood utilization [46]:

$$O=\alpha_{l}m_{l}=\alpha_{l}gM=\alpha_{l}m_{v}.$$ (18)

As described in the previous section, the larval habitat utilization rate term (*α*_*l*_) is essentially unmeasurable, and it is also very difficult to define what constitutes mosquito aquatic larval habitat in a quantifiable way [36, 38–39, 47]. Even if it is possible to quantify a sample of aquatic larval habitats (*Z*), possibly within a defined subset of habitat categories (*x*), using relatively simple indicators, such as the perimeter of the water bodies with which they are associated, it is impractical to measure directly the total quantity of aquatic larval habitat present in an entire ecosystem (*l*) on village-level spatial scales that are large enough to be epidemiologically relevant to an intervention like autodissemination that only acts at the community level [59]. However, as discussed above, it is now possible to survey oviposition events [48] as rates per sticky trap placed at a given density per perimeter length unit of habitat, even if that fixed proportion of oviposition effects that can be detected is undefined and unmeasurable [48]. Thus, it is should be possible to relate observed oviposition rates at aquatic habitats under natural conditions to those in titration experiments in which varying numbers of contaminated mosquitoes are introduced to them, following which their productivity or lack thereof is determined, so long as the same survey method is applied in both experiments. By substituting Eq 18 for *O*, the quotient of the ecosystem-wide total nightly oviposition rate by the entire adult mosquito population (*O*), divided by the total availability of all forms of aquatic habitats (*l*) or of a subset of aquatic habitats (*l*_*x*_), is

$$\frac{O}{l}=\frac{\alpha_{l}m_{l}}{l},$$ (19A)

or

$$\frac{O_{x}}{l_{x}}=\alpha_{l}\frac{m_{l_{x}}}{l_{x}}.$$ (19B)

As described in Eq 16, *α*_*l*_ *m*_*l*_/*l* can be estimated by assuming a sample of aquatic larval habitats (*l*_*z*_), or a sample of a defined subset of those habitats (*l*_*x*,*z*_) is representative, so

$$\frac{O}{l}=\alpha_{l}\frac{m_{l}}{l}=\alpha_{l}\frac{m_{l_{z}}}{l_{z}},$$ (20A)

or

$$\frac{O_{x}}{l_{x}}=\alpha_{l}\frac{m_{l_{x}}}{l_{x}}=\alpha_{l}\frac{m_{l_{x,z}}}{l_{x,z}}.$$ (20B)

Sticky traps probably under-count ovipositing mosquitoes (*ε*_*d*_<1) but the number of ovipositing mosquitoes observed in the trap can be assumed to be proportional to that absolute quantity, so the rate at which mosquitoes oviposit in a surveyed sample of larval habitats (*l*_*z*_) or subset of habitats (*l*_*x*,*z*_), that are detected by a sticky trap, may be described as

$$m_{l_{z,d}}=\varepsilon_{d}\alpha_{l}{\frac{m_{l_{z}}}{l_{z}}}_{,}$$ (21A)

or

$$m_{l_{x,z,d}}=\varepsilon_{d}\alpha_{l}{\frac{m_{l_{x,z}}}{l_{x,z}}}_{.}$$ (21B)

By rearranging Eq 29 $(m_{l_{z}}\;=\;l_{z}m_{l_{z,d}}/\varepsilon_{d}\alpha_{l}$ or $m_{l_{x,z}}\;=\;l_{x,z}m_{l_{x,z,d}}/\varepsilon_{d}\alpha_{l_{x}})$ and substituting into Eq 20, *α*_*l*_ and *l*_*z*_ or *l*_*x*,*z*_ both cancel, leaving *ε*_*d*_ as the only unmeasurable term:

$$\frac{O}{l}=\alpha_{l}\frac{m_{l_{z}}}{l_{z}}=\frac{\alpha_{l}}{l_{z}}\frac{l_{z}m_{l_{z,d}}}{\varepsilon_{d}\alpha_{l}}=\frac{m_{l_{z,d}}}{\varepsilon_{d}}.$$ (22A)

or

$$\frac{O_{x}}{l_{x}}=\alpha_{l}\frac{m_{l_{x,z}}}{l_{x,z}}=\frac{\alpha_{l}}{l_{x,z}}\frac{l_{x,z}m_{l_{x,z,d}}}{\varepsilon_{d}\alpha_{l}}=\frac{m_{l_{x,z,d}}}{\varepsilon_{d}}.$$ (22B)

Thus, Eq 22 indicates that the quotient of the ecosystem-wide total nightly oviposition rate by the entire adult mosquito population (*O*), divided by the total availability of all forms of aquatic larval habitats (*l*), may be estimated by dividing the rate at which mosquitoes ovipositing at a surveyed sample of habitats are detected with a sticky trap ${(m}_{l_{z,d}})$ by the efficiency of that trap (*ε*_*d*_), and the same applies to subsets of habitats within the ecosystem (*O*_*x*_, *l*_*x*_, and $m_{l_{x,z,d}})$, respectively).

Fortunately, the terms *O*/*l* and *T*_*l*_ appear in Eq 2 as a quotient (*O*/*lT*_*l*_), so the same applies to $m_{l_{z,d}}$ and $T_{l_{z,d}}$ or $m_{l_{x,z,d}}$ and $T_{l_{x,z,d}}$ and the unknown detection efficiency term (*ε*_*d*_) cancel in the equivalent quotient. Substituting Eq 17 for *T*_*l*_ (and then $T_{l_{x}})$, Eq 22 for *O*/*l* (and then *O*_*x*_/*l*_*x*_), we get

$$\frac{O}{l\;T_{l}}=\frac{\varepsilon_{d}m_{l_{z,d}}}{\varepsilon_{d}T_{l_{z,d}}}=\frac{m_{l_{z,d}}}{T_{l_{z,d}}},$$ (23A)

and

$$\frac{O_{x}}{l_{x}T_{l,x}}=\frac{\varepsilon_{d}m_{l_{x,z,d}}}{\varepsilon_{d}T_{l_{x,z,d}}}=\frac{m_{l_{x,z,d}}}{T_{l_{x,z,d}}}.$$ (23B)

### 2.3 Combining the model components to obtain a formulation using only field-measurable parameters

Taking Eq 2 and substituting Eq 23 for *O*/*lT*_*l*_ or *O*_*x*_/*l*_*x*_ *T*_*l*,*x*_, we get

$$C_{l}=1-e^{-\;\frac{C_{M}\;U\;m_{l_{z,d}}}{\;T_{l_{z,d}}}\;}$$ (24A)

and

$$C_{l_{x}}=1-e^{-\;\frac{C_{M}\;U\;m_{l_{x,z,d}}}{\;T_{l_{x,z,d}}}\;}$$ (24B)

where *C*_*l*_ and $C_{l_{x}}$ are the respective proportional coverage of all aquatic larval habitats (*l*) or a subset thereof (*l*_*x*_), with PPF contamination. All the parameters specified in Eq 24 that replace equivalent terms in Eq 2 are field measurable. The only term that remains from Eq 2 (*U*: the mean time that aquatic larval habitats persist but remain unproductive following contamination with PPF) also may be measured directly in the field following experimental contamination of natural habitats with at least the measured titre of live contaminated females required to render them unproductive.

## Results

Overall, Eq 24 enables prediction of larval habitat coverage with PPF contamination via autodissemination, using input parameters that are all field measurable and have a relatively straightforward deterministic relationship, so that recent successes in enclosed large-cage SFS [35] can be rationalized, and the potential for application in full field ecosystems can be assessed. Beyond merely assessing the prospects for any given PPF formulation and delivery method, the model also defines measurable properties of different prototypes that may be optimized conveniently and rapidly under controlled experimental conditions, so that prospects for success in full field ecosystems may be maximized. Furthermore, combining mathematical simulation analysis with a review of the known biological and physical constraints upon the input parameters allows assessment of the plausibility of success in full field ecosystems and threshold values or, more accurately, combinations of values for those input parameters that are required to achieve meaningful impact upon dry season malaria transmission or even the population stability of the parasite and vector populations that mediate it.

### 3.1 **Model parameterizability**

All the parameters on the right hand side of Eq 24 are, as described in the narrative of the preceding methods section, measurable not only in large-cage SFS, but also in full field ecosystems: (1) Proportional coverage of the ovipositing adult mosquito population (*M*) with PPF contamination (*C*_*M*_) may be measured directly using appropriate labels to mark insects [56] making contact with resting site surfaces that are, or would be, treated with PPF. Alternatively, it may be estimated indirectly as a function (Eq 8) of utilization rate for a defined subset of resting sites $\left({\alpha_{r}}_{x}\right)$, measured by comparing observed rates of utilization events for resting site subsets at samples of the ecosystem ${{(M}_{r}}_{x,z})$ with those for all mammalian blood resources at the same samples of the ecosystem ${{(M}_{v}}_{z})$ (Eq 9);(2) The minimum rate at which contaminated ovipositing females are captured by sticky traps placed at a defined density per unit of habitat perimeter length at a sample subset of aquatic habitats, that is required to render those habitats unproductive within one night ($T_{l_{z,d}}$ or $T_{l_{x,z,d}}$) may be estimated by titration, achieved by introducing varying numbers of contaminated mosquitoes into cages placed over those habitats, within which sticky traps are placed at a standardized density; (3) the number of oviposition events detected by the same sticky traps in the same sample of habitats under natural conditions ($m_{l_{z,d}}$ or $m_{l_{x,z,d}}$) may be measured in the same way, but with the cage removed so that it is exposed to normal levels of oviposition by the wild mosquito population $m_{l_{x,z,d}}$; and (4) The mean period of time that aquatic larval habitats persist but remain unproductive following contamination with PPF (*U*) may be measured by longitudinal observation of the habitats contaminated during the titration experiments, particularly those at the minimum effective level of mosquito exposure that defines the measured titre.

The model described by Eq 24 not only enables field parameterization, but also directly defines the design of the experiments that need to be conducted to (1) rationalize the recent demonstrations of success PPF autodissemination in enclosed large-cage SFS [35], and (2) assess the plausibility of success in full field ecosystems, using either *An*. *arabiensis*, or an alternative mosquito species with which it shares aquatic habitats, to mediate PPF transfer and coverage amplification.

### 3.2 Measurable optimization of autodissemination technologies and delivery strategies

Eq 24 also defines measurable properties of different prototype autodissemination strategies that may be rapidly optimized, often under conveniently controlled experimental conditions, to enhance prospects for success and maximize impact in full field ecosystems.

The most obvious of these is the detectable titre of ovipositing females required to render habitats unproductive ($T_{l_{z,d}}$ or $T_{l_{x,z,d}}$); while standardized artificial habitats created inside experimental cages may not be representative of their natural counterparts, nevertheless, they may be perfectly adequate and far more convenient for comparing the level of emergence inhibition activity transferred to mosquitoes by a variety of alternative PPF formulations. While such activity measurements (by definition the inverse of titre) in artificial habitats may not be used to predict likely impact in natural larval habitats, the formulation conferring the highest level of transferrable activity in such experimental systems is also probably the best option for full field application, unless some other considerations, such as persistence, acceptability, or cost are limiting.

The next most obvious parameter which might be maximized to enhance impact is the mean period of time that aquatic larval habitats persist but remain unproductive following contamination with PPF(*U*). The values for this parameter are determined and limited by the rate at which individual habitats are created and destroyed, or by the rate at which emergence activity decays in these habitats, whichever of these two rates is fastest. To a large extent, this parameter may have already been optimized to some degree simply by choosing PPF as the larvicide, because it is a relatively persistent active ingredient. However, the brief persistence times of approximately two weeks [60] for PPF in artificial habitats for immature stages of mosquitoes from the *An*. *gambiae* complex that were exposed to natural meteorological conditions and sunlight, are disappointing. The observations therefore suggest that there may yet be room to improve upon either the choice of active ingredient or its formulation, because many dry season habitats last much longer under natural conditions [36, 39, 61–62]. In advance of full field trials, it would be important to measure the actual frequency distributions of habitat and PPF persistence in natural target ecosystems to determine whether both are sufficiently long to enable adequate accumulation of emergence inhibition activity.

Furthermore, proportional coverage of the ovipositing adult mosquito population (*M*) with PPF contamination (*C*_*M*_) may be most readily and directly measured using appropriate labels to mark insects [56] making contact with resting site surfaces that are, or would be, treated with PPF [46]. In SFS, the diluting effect of the immigration of unlabelled mosquitoes upon the denominator and emigration of labelled mosquitoes upon the nominator, in the experimental results can be circumvented. However, under actual full field conditions, such an experiment would need to be conducted on a geographic scale large enough to negate the effect of mosquito dispersal [59, 63–64].

The number of oviposition events detected by sticky traps placed at samples of natural habitats ($m_{l_{z,d}})$ or subsets of habitats ${(m}_{l_{x,z,d}})$ might initially appear to be a fundamental property of the ecosystem in question. However, it is also amenable to optimization by choosing the most effective approach to PPF autodissemination in the context of local community ecology of multiple mosquito species and other vector control methods that may be applied. While all demonstrations of successful PPF autodissemination to date [29, 35] have used the target mosquito species itself to mediate PPF transfer to its own aquatic habitat, this does not necessarily have to be the case where the target species shares its aquatic habitats with others. In fact, if we examine this choice from a mathematical perspective, Eq 24 is clearly endogenous if the target species is used to mediate autodissemination because contamination coverage of larval habitats is clearly dependent upon adult mosquito density, reflected in the rate at which they are caught in sticky traps ($C_{l}↔m_{l_{z,d}}$ and $C_{l,x}↔m_{l_{x,z,d}}$). In biological terms, the impact of the autodissemination strategy will be self-limiting (${\text{lim}}_{m_{l_{z,d,0}}}{}_{\to \infty }C_{l}<1$, where $m_{l_{z,d,0}}$ is the mean rate at which ovipositing mosquitoes are captured with sticky traps at the point where the autodissemination intervention is introduced) because increasing coverage of larval habitats will progressively reduce the densities of mosquitoes that enable it, unless either (1) larval populations of the target species are eliminated (*C*_*l*_→1)before the adult population driving it die off, and PPF contamination of those habitats persists longer than that remaining adult population so that re-infestation is prevented, or (2) a different mosquito species is used to mediate PPF transfer that co-occupies most of the target species habitats simultaneously or before the target species. Such a non-target mosquito species for autodissemination of PPF to target species aquatic habitats should also ideally behaviourally and/or physiologically resilient to control with other vector control measures that may be present, such as LLINs and IRS, so that it persists and oviposits at high densities (maximum $m_{l_{z,d}}$) even as autodissemination progressively controls, and ideally eliminates [6], the target species.

### 3.3 Minimum threshold value combinations for measurable input parameters to render intervention impact plausible

**Fig 2:**
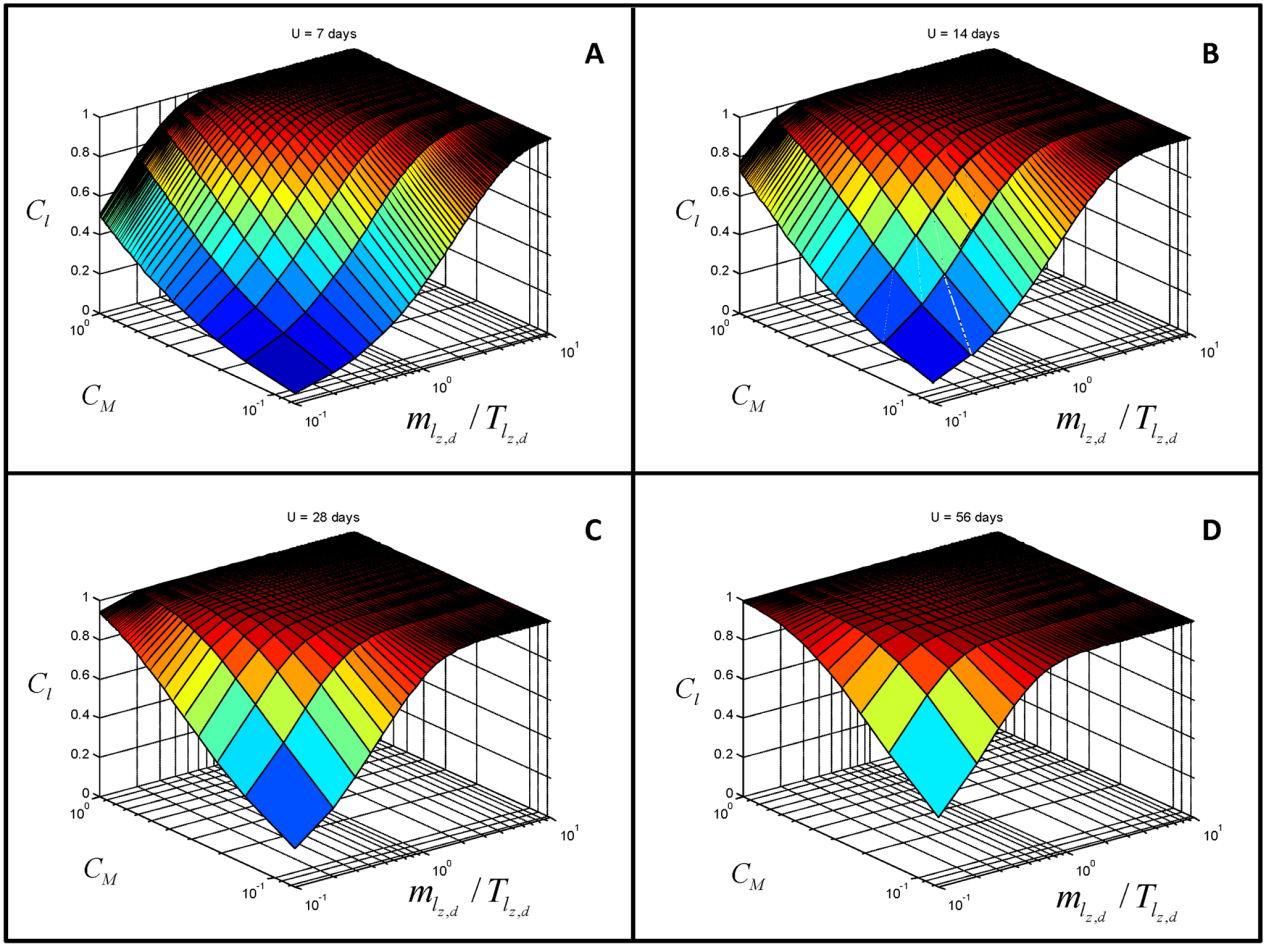
Evaluation of the proportion of all larval aquatic habitats which are effectively contaminated with PPF *C*_*l*_ at different values for the mean time that habitats persist but remain unproductive (*U*). This figure presents combinations of values for proportional coverage of the ovipositing adult mosquito population with PPF contamination *C*_*M*_, ovitrap-detectable rates of oviposition by wild mosquito (*m*_*l*,*z*,*d*_), and the titre of contaminated mosquitoes required to render habitats unproductive (*T*_*l*,*z*,*d*_) that may lead to specific values of *C*_*l*_ at different values of *U*.

If aquatic larval habitats are assumed to be created, destroyed, and replaced weekly, or that PPF activity lasts only a week in natural habitats [60] (*U* = 7 days), the minimum target of 90% coverage of aquatic larval habitats with PPF may be achieved if the proportional coverage of the ovipositing adult mosquito population (*M*) with PPF contamination (*C*_*M*_) is at least 0.33 and the quotient of the ovitrap-detectable rates of oviposition by wild mosquitoes in natural aquatic habitats contact divided by the titre of contaminated mosquitoes required to render them unproductive ${(m}_{l_{z,d}}/T_{l_{z,d}})$ approaches unity (Fig 2A). If aquatic larval habitats and PPF are assumed to persist for longer periods, the required thresholds of *C*_*M*_ and $m_{l_{z,d}}/T_{l_{z,d}}$ are less stringent and 90% contamination coverage of larval habitats with PPF may be achieved if values for all these determinants are considerably lower (Fig 2B–2D). For example, values of only 0.3 for both *C*_*M*_ and $m_{l_{z,d}}/T_{l_{z,d}}$ may be sufficient to contaminate 90% of habitats that persist and retain PPF activity for approximately one month (Fig 2C).

While infinite possible combinations of values for *C*_*M*_, $m_{l_{z,d}}$, and $T_{l_{z,d}}$ exist that can result in a given level of predicted larval habitats coverage with PPF contamination (*C*_*l*_), the apparent complexity of inputs and outputs illustrated by the responses surfaces in Fig 2 follow remarkably simple relationships: All the panels of Fig 2 are symmetric because of the simple multiplicative relationship between $C_{M}\;and\;m_{l_{z,d}}/T_{l_{z,d}}$ in Eq 24. In fact, the titre of contaminated mosquitoes required to render natural habitats unproductive appears in Eq 24 as its reciprocal $1/T_{l_{z,d}}$, which is mathematically equivalent to the activity (*A*) of contaminated mosquitoes, so any increase in one of these terms can compensate exactly for a proportional decreases in the others, with the caveat that *C*_*M*_ is a proportion and therefore constrained to values of less than one. Their combined effect can be represented as a direct function of their product, leaving the question as to how these three parameters may be optimized to achieve predicted threshold values for their product as an open matter for debate, experimentation, and measurement.

**Fig 3:**
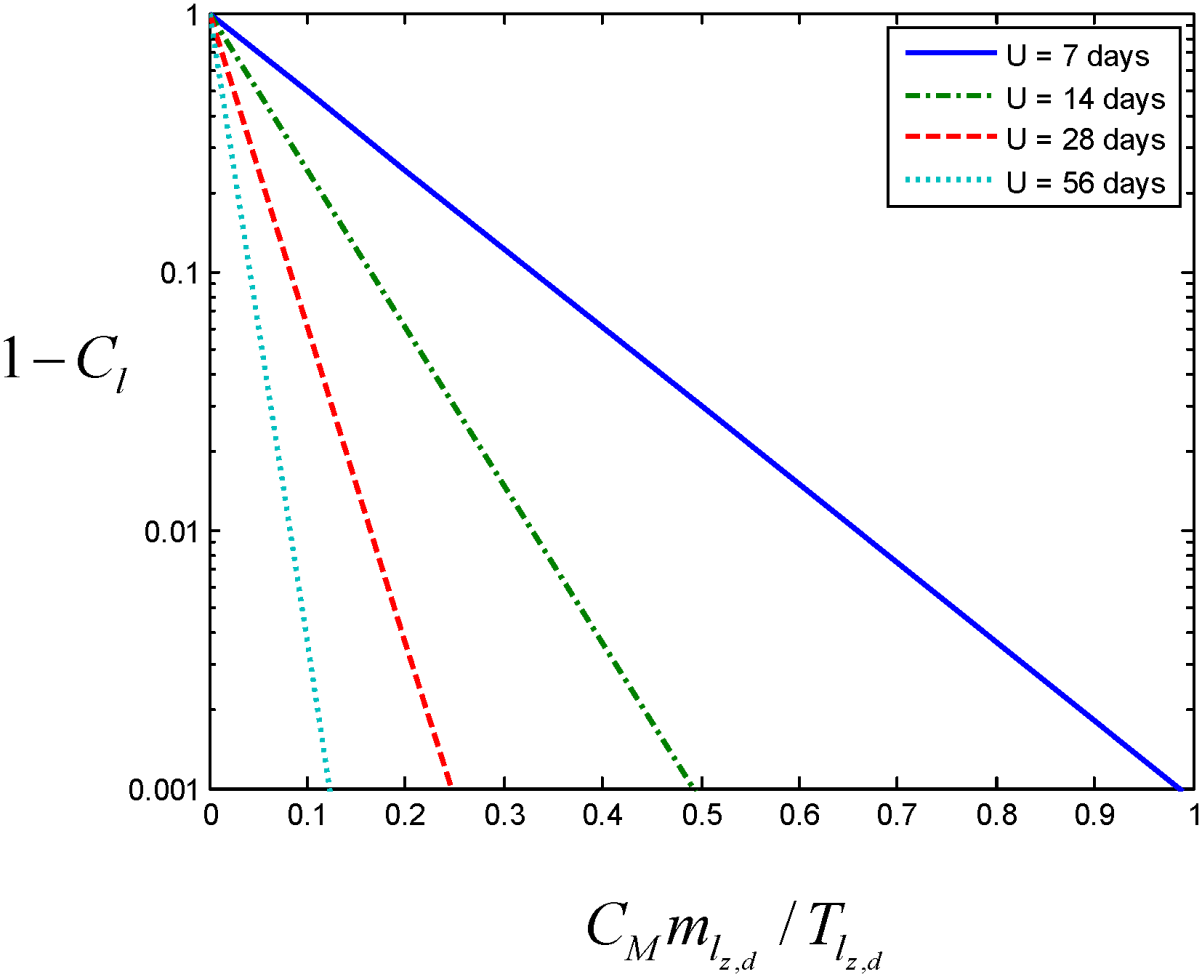
An illustration of the main three input parameters in predicting the proportion of all aquatic habitats contaminated with PPF. This figure presents combined influence of *C*_*M*_, $m_{l_{z,d}}$, and $1/T_{l_{z,d}}$ upon the availability of uncontaminated aquatic habitats (1-*C*_*l*_) to the vector population as a simple exponential decay, so that the increasing threshold values required to achieve specific larval habitat coverage targets can be visualized as a log-linear function of their product.

Furthermore, because the autodissemination strategy is limited in applicability to the dry season, and essentially needs to eliminate malaria transmission [40], or even the vector population itself [6], before the rains return to be worthwhile, so very ambitious larval habitat coverage targets must be set (*C*_*l*_ > 99%). Just like field measurement of progress in any elimination programme [65], visualizing simulated progress towards zero requires a corresponding change in perspective and scale. Fortunately, the combined influence of *C*_*M*_, $m_{l_{z,d}}$, and $1/T_{l_{z,d}}$ upon the availability of uncontaminated aquatic habitats (1-*C*_*l*_) to the vector population is described by Eq 24 as a simple exponential decay. Therefore, the increasing threshold values that are required to achieve these more ambitious larval habitat coverage targets can be visualized as a log-linear function of their product (Fig 3).

Any autodissemination intervention aiming to eliminate malaria transmission or vector populations needs to achieve comprehensive coverage of essentially *all* habitats (1-*C*_*l*_ < 0.01). Prospects for success at this high level of ambition will be limited by the most challenging, presumably ephemeral, of the subsets of targeted aquatic habitats, and recent studies from Kenya suggest that PPF activity may also not last much longer than a week [60]. The predicted threshold values for the product of *C*_*M*_, $m_{l_{z,d}}$, and $T_{l_{z,d}}$ for such short-lived habitats and insecticides (*U*_*x*_ = 7 days) therefore probably represent the most appropriate targets for optimizing and evaluating prototype autodissemination strategies based on field measurements of these three input parameters. As illustrated in Fig 3, values for $C_{M}m_{l_{z,d}}/T_{l_{z,d}}$ or ${C_{M}m}_{l_{x,z,d}}/T_{l_{x,z,d}}$ that approximately approach unity will be required to achieve at least 99% contamination coverage of this most challenging subset of habitats.

## Discussion

The greatest strength and weakness of this model is its simplicity. The simple multiplicative relationship between *C*_*M*_ and $m_{l_{z,d}}/T_{l_{z,d}}$, and the fact that their combined effect can be described as a simple exponential decay of uncontaminated aquatic habitats, allows ready application of this model by theoreticians and field biologists alike. The most important caveats and limitations to applying this model relate to uncertainties about the validity of the underlying simplifying assumptions and the natural or achievable ranges of its input parameters. Specific details and caveats we have deliberately chosen not to explicitly incorporate into the mathematical formulation presented here include: (1) Quantification of habitat size in absolute volumetric terms, and the transfer of PPF in terms of absolute dissolved concentration; (2) The endogenous feedback loop between autodissemination processes and their impact if the target mosquito species itself is used to deliver the PPF to aquatic habitats; (3) The latent effects of PPF upon adult mosquitoes exposed to sub-lethal doses as larvae [60] and; (4) The temporally dynamic and spatially complex nature of both adult and juveline mosquito populations, as well as malaria transmission.

No attempt was made to capture the detailed processes of PPF adsorption and delivery to aquatic habitats by mosquitoes in terms of mass quantities, or to calculate its concentration in solution following dilution into water bodies of defined volumetric size. While such explicit physico-chemical model components might be attractive in principle, they would probably only be practically useful if the amount of PPF carried and released by individual mosquitoes could be readily estimated and related to specific volumes of water bodies that can be contaminated. While the former is technically challenging at the very least, the latter is essentially impossible for the diverse, dynamic and poorly-defined natural aquatic habitats of mosquitoes from the *An*. *gambiae* complex [36, 38–39]. Also, such a chemically explicit approach would need to assume that PPF activity occurs only in solution, without any interactions with the various solutes and insoluble solid substrates or particulate matter that occur ubiquitously in real *Anopheles* habitats. Indeed, in our latest demonstration of successful autodissemination by *An*. *arabiensis* in large cages, the PPF activity exhibited by the contaminated habitats themselves appeared to be far greater than that of the water when it was removed for separate bioassay without the mud substrate [35]. The common biochemical approach of measuring and expressing effective activity of an agent under relevant conditions, rather than as mass quantity, was therefore adopted. Biochemical or biological activity can be expressed as the simple inverse of titer (*A = 1/T*), which can in turn be measured directly by titration. The measurable titer of detected oviposition events per sticky trap required to render a habitat or sample of habitats unproductive $(T_{l_{z,d}}$ or $T_{l_{x,z,d}}$) is therefore directly included in this model, conveniently yielding a quotient by dividing into their natural detectable rates of oviposition input per unit of perimeter length ($m_{l_{z,d}}$ or $m_{l_{x,z,d}}$), so that the common unknown fractions of all oviposition events which are detected by both measurements cancel each other out (Eq 23).

Perhaps the most obvious limitation of this model is that it ignores the endogenous relationship that occurs between the output parameter and one of the input parameters if the target mosquito species is used to mediate PPF transfer: In simple terms, the success of the autodissemination strategy requires availability of gravid *Anopheles* mosquitoes to transfer PPF, but accumulation of PPF in aquatic habitats will inhibit emergence of those same mosquitoes. While incorporating delivery-impact endogeneity upon an *Anopheles* population which was both the target and mediator of autodissemination, would have improved the realism with which such an attempted application could be modelled, we have instead chosen to retain this feature so we could illustrate how such a strategy could be self-defeating. This flaw is deliberately retained in the model, not only because it allows dramatic, parsimonious simplification, but also because it lucidly illustrates the potential advantages of using a different mosquito species that shares the same aquatic habitats as the primary target for contamination at selected resting sites. For example both *An*. *arabiensis* and *An*. *gambiae* larvae typically share habitats with a variety of other *Anopheles* and far greater numbers of diverse *Culicine* species [36,39,66] so it may well be feasible to use these non-target species as vehicles for mediating autodissemination. Of course, using such non-target species as autodissemination vehicles may also be recursive or endogenous, but will nevertheless probably mediate higher sustained levels of PPF delivery than using the target *Anopheles* species, so long as they naturally outnumber them in their shared habitats under baseline conditions.

A number of factors prompted us not to explicitly capture the impact of sub-lethal doses upon the fecundity of adults exposed as larvae: (1) These sub-lethal effects occur over a relatively narrow range of concentrations relative to the logarithmic scales of parameter space explored here, with the doses that cause sub-lethal effects closely approaching those causing none because adult emergence is entirely inhibited [60]; (2) As explained above, the specific application of this approach as a dry season tool for elimination, rather than control, of malaria transmission [40–41] or even the vector population itself [6], necessitates very aggressive coverage targets; (3) The considerable uncertainties regarding the simplifying assumptions and parameters value ranges of even this simple model are therefore most probably far greater in magnitude than the contribution of these sub-lethal effects to overall impact; (4) Capturing such a minor component of overall impact would not be worth the additional model complexity. Indeed, a more detailed model would probably be prohibitively complex to be experimentally validated or worthwhile as a tool for planning and justifying large-scale field trials.

A variety of empirical field studies [42, 66–67] and theoretical simulation analyses [37, 68–71] have illustrated just how important the dynamism and spatial complexity of malaria transmission and vector population dynamics can be. The dispersal of gravid female mosquitoes from resting sites to oviposition sites, and the choices they make in selecting where exactly to deposit their eggs, can influence the distribution of both mosquito larvae and the autodisseminated PPF required to stifle their emergence as adults, as well as where subsequent malaria transmission occurs [37, 66–69, 71]. Capturing the abundance, diversity and dynamism of all the resources that mosquitoes use is further complicated by the fact that most of them cannot be realistically classified into distinct categories that represent homogeneous systems. For example, the persistence, or required titre, of PPF in aquatic habitats may not fall into natural groupings but rather represent a continuum. All these factors and processes are very heterogeneous and wildly dynamic in real malaria-endemic ecosystems so a purely deterministic model like the one presented here, that assumes homogenous interactions between mosquitoes and their environment within a closed systems, can only capture and represent ecosystem-wide mean values that mask several subtle but important phenomena that arise in more realistic, heterogenous systems, particularly in relation to larval source management strategies [37, 68–69]. The development of autodissemination as a malaria control strategy can therefore benefit from simulation analyses using such advanced, more complex models to evaluate impact plausibility and optimize technical approaches to maximize impact. However, the complexity of such models is inevitably associated with disadvantages as well as advantages, and is therefore complementary to the simple, parsimonious, field-parameterizable formulation developed and presented here, rather than a substitute for it.

Perhaps the best example of how detailed consideration of the environmental and biological processes can allow the formulation of parsimonious solutions arises from the observation that the hydrological systems that support aquatic larval habitats approach steady-state conditions in the dry season [38, 68] when the autodissemination strategy may be optimal for reasons detailed in the introduction. The model described by Eq 24 and the numerous applications described below all depend on the implicit assumption of steady-state conditions, despite the fact that African malaria vector populations are often considered to be highly dynamic, especially those of species from the *Anopheles gambiae* complex to which *An*. *arabiensis* belongs [36, 39, 42]. However, recent field observations [38] examining the hydrology of malaria in the particularly well-characterized village of Namwawalla in rural southern Tanzania, confirms that for 2 to 3 months of the dry season, essentially all larval habitat is continuously created and then destroyed by the receding groundwater table. The total quantity of aquatic habitat remains approximately stable but reflects a constant turnover of habitats with life-spans of days and weeks as the perimeter of water bodies recedes along varying gradients, even though many of the water bodies they are associated with may last for months [38]. The spatial distribution of optimal habitat across populations of depressions in the landscape varies from week to week as their shallowest fringes are first exposed and then drained by the dropping water table. Therefore, this can be treated as an example of a system of larval aquatic habitats and associated mosquito populations that are dynamic, but nevertheless approximate steady-state conditions. The parameters of Eq 24 may therefore, all be measured and reasonably used to predict impact of autodissemination strategies during the depth of the dry season from August to October. Other studies of dry season larval habitat ecology for members of the *Anopheles gambiae* species complex describe larval habitat dynamics that are at least as stable and provide several examples of where permanent or semi-permanent habitats are seasonally important during such annual minima of larval habitat availability and mosquito population density [36, 39, 61–62]. Bearing in mind the limitations of any mathematical model, defined as a deliberately simplified representation of complex real world processes expressed in mathematical terms, Eq 24 may be applied to optimize prototype approaches to PPF autodissemination, and to assess the plausibility of success for specific approaches, based on field measurements of its input parameters.

So, while acknowledging the inevitable limitations of the parsimonious, measurement-oriented approach deliberately taken here, the remarkably simple formulation described in Eq 24 also has considerable advantages. All required inputs can be measured in the field, so recent successes in enclosed large-cage SFS may now be rationalized using this relational framework, and the plausibility of success under full field conditions can be evaluated *a priori* based on direct measurements in potential field sites. The model also defines measurable properties of different prototypes that may be conveniently and rapidly optimized under controlled experimental conditions to maximize chances of successful application at ecosystem scale in full field trials.

## Conclusions

For autodissemination interventions to eliminate malaria transmission or vector populations during the dry season window of opportunity will require comprehensive and sufficient contamination of all aquatic habitats (*C*_*l*_→1), including the most challenging subset of these that persist or retain PPF activity for as little as a week ($C_{l_{x}}\to 1$, where *U*_*x*_ = 7 days). The model presented here suggests that to achieve at least 99% contamination coverage of this ephemeral aquatic habitats subset will require values for the product of the proportion of the mosquito population that is contaminated multiplied by ovitrap-detectable rates of oviposition by wild mosquito into this subset of habitats, divided by the titre of contaminated mosquitoes required to render them unproductive, to approach unity $\left({C_{M}\;m}_{l_{x,z,d}}/T_{l_{x,z,d}}\to 1\right)$.
